# Custom Array Comparative Genomic Hybridization: the Importance of DNA Quality, an Expert Eye, and Variant Validation

**DOI:** 10.3390/ijms18030609

**Published:** 2017-03-10

**Authors:** Francesca Lantieri, Michela Malacarne, Stefania Gimelli, Giuseppe Santamaria, Domenico Coviello, Isabella Ceccherini

**Affiliations:** 1 Dipartimento di Scienzedella Salute, Sezione di Biostatistica, Università degli Studi di Genova, Via Pastore 1, 16132 Genoa, Italy; 2 Struttura Complessa Laboratorio Genetica Umana, E.O. Ospedali Galliera, Via Volta 6, 16128 Genoa, Italy; michela.malacarne@galliera.it (M.M.); domenico.coviello@galliera.it (D.C.); 3 Department of Medical Genetic and Laboratories, University Hospitals of Geneva, Bâtiment de Base 8C-3-840.3, 4 Rue Gabrielle-Perret-Gentil, 1211 Geneva 14, Switzerland; stefania.gimelli@gmail.com; 4 UOC Genetica Medica, Istituto Giannina Gaslini, L. go G. Gaslini 5, 16148 Genoa, Italy; giuseppesantamaria@gaslini.org (G.S.); isa.c@unige.it (I.C.)

**Keywords:** high density custom CGH array, agilent aberration call software, CNV detection filters

## Abstract

The presence of false positive and false negative results in the Array Comparative Genomic Hybridization (aCGH) design is poorly addressed in literature reports. We took advantage of a custom aCGH recently carried out to analyze its design performance, the use of several Agilent aberrations detection algorithms, and the presence of false results. Our study provides a confirmation that the high density design does not generate more noise than standard designs and, might reach a good resolution. We noticed a not negligible presence of false negative and false positive results in the imbalances call performed by the Agilent software. The Aberration Detection Method 2 (ADM-2) algorithm with a threshold of 6 performed quite well, and the array design proved to be reliable, provided that some additional filters are applied, such as considering only intervals with average absolute log_2_ratio above 0.3. We also propose an additional filter that takes into account the proportion of probes with log_2_ratio exceeding suggestive values for gain or loss. In addition, the quality of samples was confirmed to be a crucial parameter. Finally, this work raises the importance of evaluating the samples profiles by eye and the necessity of validating the imbalances detected.

## 1. Introduction

Array-based comparative genomic hybridization (aCGH) has provided a new impulse to cytogenetic diagnostics and has proved to be a valuable tool in the clinical management of patients with developmental delays and multiple congenital anomalies [1]. This approach has also allowed the identification of novel chromosomal syndromes [2,3], helped to define the clinical variability associated with several genomic disorders [4], and led to the discovery of polymorphic copy number variants (CNVs) in the human genome [5,6,7].

Early CGH arrays were composed of large-insert bacterial artificial chromosome (BAC) clones and later evolved to microarray-based technology oligoarray CGH (aCGH).

There were concerns about procedure variability and interpretation criteria for the clinical application of early versions of targeted BAC clone array because of the presence of false negative results [8]. Two studies attempted to estimate the false positive (FPR) and false negative (FNR) rates. Wong et al. [9] analyzed six repeated experiments on 95 individuals. Given the very low binomial probability of detecting by chance the same clone twice within six experiments, they assumed that any clone detected twice or more in their experiments was a true CNV [9]. In this way, they calculated a FNR of 45.3% and a FPR of 0.23%. Following a similar method, de Smith et al. [10] calculated an estimate of FNR of 0.16 [10]. The FPR was instead estimated to be 0.05 for multi-probe calls by using three self-self hybridizations of the reference sample and comparing the average number of variant interval calls with that calculated for each sample. However, a few studies validated BAC clone arrays by examining well ascertained CNVs [11,12].

The more recent development of oligonucleotide aCGH has led to a greater resolution in CNV identification. Concordance for oligo aCGH with BAC array was shown [13,14], with around 99% sensitivity and 99% specificity [11,15] and superior performance of oligonucleotide aCGH over BAC clone aCGH [13,16].

However, all these studies based their confirmatory results on imbalances of standard cytogenetic size (Mbs). In addition, high probe density might generate more noise in aCGH data. Using two different Agilent CGH microarrays, it has been shown empirically that subject-to-subject variance is almost twice as large as array-to-array and dye-to-dye variance, supporting results reliability. However, the same study showed that the array-to-array variability was more than 10 times larger than both subject-to-subject and dye-to-dye variance for a custom microarray [17]. This observation was suggested to be ascribable to the fact that to achieve the highest possible density coverage might have lead to include less reliable probes.

The use of oligo array, though increasing resolution, has lead to a lower specificity and higher potential for noise, with need for several adjacent probes to confidently identify CNV regions and a large amount of data analysis and result interpretation. Each of the necessary steps of data transformation, normalization and summarization involves algorithm parameters that directly affect the sensitivity and specificity of the aCGH assay and represents a source of potential Type I and Type II errors [18]. The analysis can be complicated by the presence of platform and method artifacts including GC-waves [19,20] and by centralization methods [21].

More recently, the growing use of custom arrays, which are based on libraries of validated synthetic probes that can interrogate relevant genomic regions, have further enhanced the resolution capabilities of targeted regions [22,23,24,25,26]. In addition, single nucleotide polymorphism (SNP) arrays have been exploited to search for CNVs: not feasible for single-exon resolution throughout the genome, they nonetheless have the advantage to provide genotypes and detect regions of absence of heterozygosity (AOH) thus also allowing the identification of uniparental isodisomies (UPD) and genetic identity by descent [27]. However, the amount of data produced by SNP arrays is computationally challenging and requires a burden of analysis and filters to allow interpretation of results, and it is not yet well described whether the widely used SNP-array-based CNV calling methods can provide sufficient concordance with CGH in CNV detection [28]. In addition, SNP array were shown to not outperform oligonucleotides aCGH in a study that carried out a CNV search by the Affymetrix 6.0 SNP array on patients with developmental disorders already found negative by oligo aCGH at higher resolution [29]. Combining SNPs and oligo arrays in a single assay is increasingly being employed, with the advantage to obtain genotypes and a higher resolution with respect to aCGH data alone [30]. However, if the final goal is limited to the search of CNVs, oligo aCGH remains the most cost effective and straightforward method.

Several quality metric variables can be used to evaluate the quality of the oligo array and dataset, such as probe-to-probe log_2_ratio noise, signal intensity, background noise channels, signal to noise and reproducibility. In addition, CNVs are generally claimed when several probes are indicative of CNVs, although this reduces the array resolution. However, false positive and false negative results are still an issue.

A few studies regarding preimplantation genetic screening in human assisted reproduction showed a not infrequent presence of false positive and false negative results from aCGH [31,32]. In particular, Capalbo A. et al. [31] compared aCGH and qPCR on 120 aneuploid blastocysts, finding that 18.3% of embryos gave a discordant result for at least one chromosome and that most of these were due to aCGH false positive results.

A small number of other studies mention the presence of false positive and false negative results detected, for instance, by the use of different aberration detection methods [24], or the finding of possible aberration missed at aCGH when reanalyzing the results by Next Generation Sequencing (NGS) [26]. However, in our opinion, the presence of false and positive results is not addressed enough in the results reported in the literature.

Abnormal results should be confirmed and various strategies have been described to follow-up analysis, including repeated aCGH testing, fluorescence in situ hybridization (FISH), microsatellite analysis, multiplex ligation-dependent probe amplification (MLPA) and, in particular, real-time quantitative PCR (qPCR). Recently, NGS has also been suggested [26].

Nonetheless, aCGH remains the first-tier testfor CNV detection, due to its genome wide applicability. Validation on a large number of patient and control samples following aCGH analysis is not practical, and it is not always reported in large screenings [33,34], or only few interesting candidates are validated [26].

We took advantage of a custom aCGH, recently carried out on 59 patients affected by Hirschsprung Disease (HSCR) to search for imbalances in genes and loci candidate for HSCR [35], to analyze its performance, the use of several aberrations detection algorithms, and the presence of false positive and false negative results.

## 2. Results

### 2.1. Sample Quality and Design Reliability

**Figure 1:**
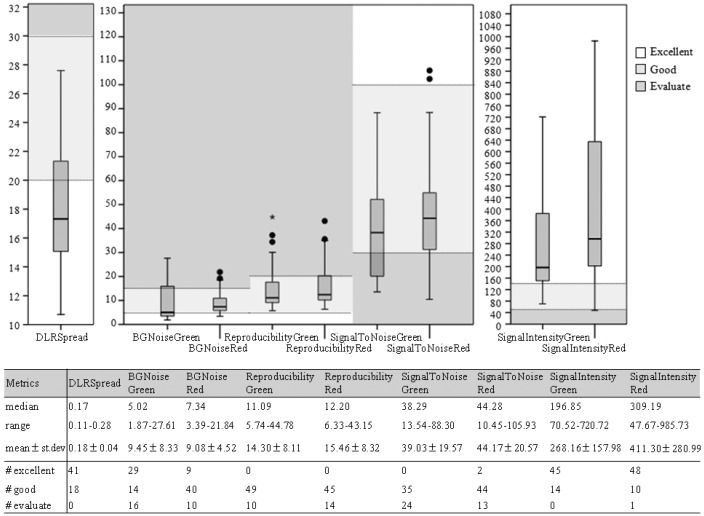
Quality control metrics: Distribution of the sample quality controls is reported as box plots and as statistics. In particular, sample metrics are highlighted as excellent, good or poor (evaluate) and how many samples are in each category is also reported. Solid circles and asterisks in the box plot graphs represents the outliers: solid circles are cases with values more than 1.5 times the InterQuartile (IQ) range, asterisks are cases with values more than 3 times the IQ range.

DLRSs (derivative log ratio spread) and the other metrics of the final 59 samples analyzed for the search of aberrations are shown in Figure 1. The DLRS, in particular, is a measure of the log ratio noise for each sample, calculated as the standard deviation (spread) of the log ratio differences between consecutive probes.

To assess the reliability of the results, we correlated the log_2_ratios between replicates as reported elsewhere [36], finding low correlations (mean *r* = 0.18 across 37 comparisons), though higher than among random sample pairs (mean *r* = 0.07, *p* = 0.0040) (Table 1). Such a low correlation is not unexpected since log_2_ratios not different from zero are supposed to vary randomly. As a matter of fact, considering only log_2_ratios with absolute values above 0.3, the mean correlations improved in both replicated and random sample pairs (mean *r* = 0.42, *p* = 1.8 × 10^−9^ and 0.14, *p* = 0.0036, respectively), but at a much higher rate for the replicates (*p* = 4.8 × 10^−5^). These results reassure on the design quality and suggest that the application of a minimum log_2_ratio values criterion, such as the mean absolute log_2_ratio > 0.30 (hereafter referred as MALR > 0.30), is reasonable and advisable. Not surprisingly, the level of correlation is dependent on the derivative log ratio spread (DLRS): correlations between replicates and random pairs were significantly different only when at least one sample had DLRS ≤ 0.2 (*p* = 6.8 × 10^−6^ on |log_2_ratio| ≥ 0.3). Correlations were much lower and not significant for the 13 pairs with both samples with DLRS > 0.2 (*r* = 0.21 for replicates and *r* = 0.09 for random pairs).

Of note, although we could not find any correlation between the DLRS and the log_2_ratio values, besides a faint negative trend, correlations between pairs in which at least one sample had DLRS ≤ 0.2 were significantly higher than correlations between pairs without any sample with DLRS ≤ 0.2, for replicates but not for random pairs (i.e., *r* = 0.53 vs. 0.21, *p* = 0.0009 for replicates and *r* = 0.17 vs. 0.09, *p* = 0.2188 for random pairs on |log_2_ratio| ≥ 0.3) (Table 1).

Following the method described elsewhere [10], we estimated the FPR for the Aberration Detection Method 2 (ADM-2) at threshold 6 to be 0.130 for single-probe calls and 0.184 for single-probe calls, higher than that estimated by de Smith et al. [10]. However, all the aberrations called in the three self-self test regarded the high density region around *RET* (10q11.2), confirming that this sub-centromeric region is problematic, and presented with MALR < 0.3, so that applying such a filter the FPR virtually dropped to 0. Of note, all the calls on this gene, including those that seemed likely or possible, were not confirmed at validation.

We also estimated the FNR in a manner similar to that described by Wong et al. and de Smith et al. [9,10]. In the four replicated experiments, 13 putative variant intervals were observed twice and then considered true calls, yielding an estimate of FNR of 0.50. We also evaluated three samples assayed in triplicate, finding an average FNR of 0.213. The FNR found in the four replicates experiments was slightly higher than that estimated by Wong et al. [9] and definitely higher than that estimated by de Smith et al. [10], while it was closer to their estimates for the three replicates experiments. However, we calculated that if Wong et al. [9] had performed four (or three) replicates, they would have estimated a FNR of 0.3629 and 0.2111, respectively, similar to those obtained by us.

The studies by both de Smith et al. [10] and Wong et al. [9] made use of BAC-based CGH, so that a direct comparison with our results might be misleading. Nevertheless, a high FNR (above 20%), with calls that were missed in one or more replicates, and a very low FPR were confirmed. This latest estimate is based on a reference DNA with very high quality (DLRS < 0.15) and we cannot exclude that FPR would have been higher with worse quality samples. Accordingly, few imbalances could not be validated or confirmed in replicates.

### 2.2. High Density Design Performance

**Figure 2:**
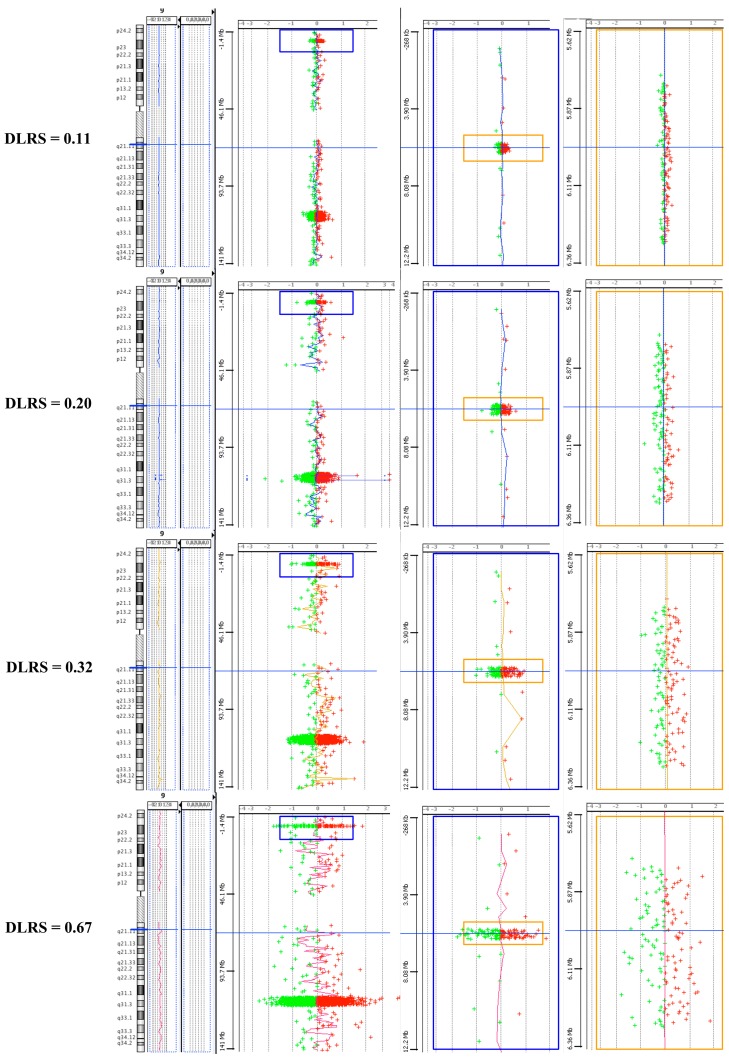
Sample profiles. An example of four samples selected for excellent quality, good, evaluate and very bad quality. For each, the profile at chromosome 9 is shown, including a region of probes scattered across the genome and two high density regions. The upper high density region inside the blue box in the left panel is zoomed in into the central panel (inside the large blue box) and the specific region inside the yellow box is further zoomed in into the right panel (inside the large yellow box).

Notwithstanding the possibility that high density regions might present low replicability and display worse profiles than less high density region, when zooming in the regions we did not observe any higher variability in the profiles between high density probes regions and the rest of the genome (Figure 2).

**Figure 3:**
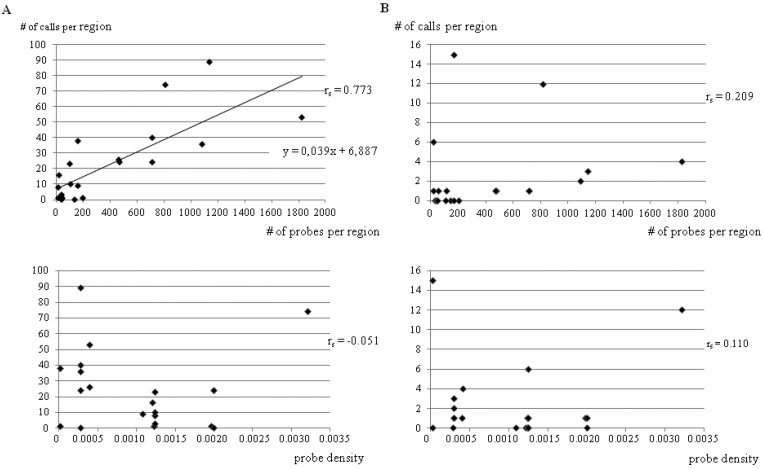
Aberration calls and probes correlations. Correlation between the number of calls detected in each high density region and the number of probes selected in each region (**upper**) or the probe density (number of probes/size) of the selected region (**bottom**) considering any calls, including: single probe calls (**A**); or only multi probes calls with MAAD > 0.3 (**B**).

Accordingly, we found that the number of calls in the selected regions correlated with the number of probes analyzed (*r* = 0.773) as expected, while it did not correlate with either the size or the probe density of the region selected. These observations were confirmed considering only calls sustained by at least two probes or considering the number of probes called instead of the number of calls. When only calls with MALR > 0.3 were considered, no correlation could be detected at all (Figure 3).

Redundancy of probes covering the same target sequence might instead create problems in the hybridization and thus false results, as we could observe in a preliminary design.

### 2.3. Comparison between Algorithms and Filters

Applying the ADM-2 algorithm with a threshold of 6, the CG correction and the centralization algorithms, and excluding the positive control regions, we got 572 aberration calls on autosomal chromosomes (for a median of seven calls per sample, range 1–34), 393 of which sustained by at least two consecutive probes (five median calls per sample, range 1–17). We also repeated the analysis without the GC correction but no difference could be detected in the results.

However, at the samples profiles visual inspection, most of the calls seemed to us as false positives, for this reason we have also applied two additional filters, MALR > 0.30 and threshold_e_ > 0.33. With the first filter we obtained 75 calls, six of which corresponding to the already known chromosomal alterations (for these latter we got nine calls, but five were overlapping for the two chromosomal *RET* deletions on the two sample used as controls for this region and are not reported in Table 2). Two CNVs were not found in the best quality sample firstly evaluated but were added to the list because found in the two additional good quality replicates. These 77 aberrations reduced to 52 considering the threshold_e_ > 0.33, an empirical threshold based on the number of probes with specific log_2_ratio ranges that we have applied to the present data (see the methods) (Table 2).

Forty-two calls were detected with the same two filters applying the Fuzzy-zero algorithm, while 22 aberrations were called applying the ADM-2 algorithm with a more stringent threshold of 8.

The visual inspection of the sample profiles allowed us to add six calls to the list of aberrations, for a total of 83 aberrations in 61 different chromosomal locations (including controls) in 44 samples.

Excluding aberrations previously reported on DGV and controls, 51 aberrations were detected in 25 patients, two of which repeated in three patients each. The variants called reduced to 25 aberrations in 17 patients when applying the threshold_e_ > 0.33, to 15 aberrations in nine patients applying the more stringent threshold of 8 and to 24 aberrations in 16 patients applying the fuzzy-zero algorithm (Table 2).

For 15 of the novel aberrations we had at least one replicate sample available, although for two samples the replicate was of low quality (DLRS ≥ 0.3). While one replicate was inconclusive because of a too noisy profile (a low quality replicate), six were definitely not replicated, four were likely although not called by the software and four were clearly replicated, although two showed a different size.

### 2.4. Software Algorithms Calls and Visual Inspection

The visual evaluation of the log_2_ratio sample profiles allowed the identification of additional putative aberrations, not detected by the software, suggesting the substantial chance of false negative results. Of the 44 CNVs classified as likely or possible based on visual inspection and further verified, 39 resulted to be true (confirmed at the validation, detected also on a second replicate or reported on DGV and thus assumed as true, in addition to the six known aberrations all very well visible). Among those classified as unlikely, instead, only four could be claimed as true while eight were excluded at the validation (*p* = 0.0004), suggesting that the visual inspection of the sample profile is crucial, and even more reliable than the use of algorithms for the variant calls (Table 3).

To note, by comparing the visual inspection results with the software calls under various scenario (a more stringent threshold of 8 for the ADM-2 algorithm, the application of the Fuzzy zero algorithm and threshold_e_ > 0.33 filters), under the application of the MALR > 0.3 with at least two probes filter, the ADM-2 algorithm with threshold 6 together with the application of the threshold_e_ > 0.33 filters resulted to be the most comparable with the visual inspection, and the threshold_e_ > 0.33 in general resulted to be a good discriminatory filter (Table 3). Both the ADM-2 algorithm with a threshold of 8 and the Fuzzy zero algorithm missed too many true calls (25 and 15, respectively).

## 3. Discussion

The search for CNVs in genes and loci candidate for HSCR in a panel of individuals affected by the disease has provided the opportunity to investigate in more detail the quality of our custom aCGH design and to address some general remarks. First of all, we could confirm that the use of a high density design does not seem to increase the error in variants detection, neither lead to worse sample profiles, thus confirming the validity of this strategy of searching for small imbalances, otherwise undetectable.

The size of the imbalances that can be detected depends upon the density of probes targeting the regions of interest and the criteria set for software-generated calls (i.e., minimum two probes as applied here). Redundancy of probes covering the same target sequence might create problems in the hybridization and thus false results. In addition, not all probes perform equally well. However, the selection of probes and the density in the coverage of the array, which in our design was up to one probe every 250 nt (for *RET*), might greatly increase the CNV search resolution, provided that there is no probe overlap. We could detect a few imbalances that are less than 5 kb that were successfully confirmed by other techniques.

Not surprisingly, the quality of samples is confirmed to be a crucial step. There is no correlation between the number of calls detected by the algorithm software and the DLRS of the samples, as it is expected since the algorithm already took into account the sample quality. However, the log_2_ratio correlation between replicates was greatly improved when DLRS were excellent (≤0.2). To note, among calls with MALR > 0.3, those detected in samples with excellent DLRS were classified as “likely” or “possible” more frequently than those detected in sample with worse DLRS. “Likely” and “possible” variants could be grouped, although “likely” variants seemed to be true variants more often than “possible” variants (30 vs. 4 compared to 4 vs. 1).

Above all, our study highlighted the importance of the visual inspection by an expert eye. The human eye can take into account several factors such as the general profile of the sample, the specific region profile, and the single log_2_ratio values involved in the putative aberration qualitatively better than any algorithm. Of course, in the case of a large screening, the use of algorithms becomes a pivotal tool. In this case, we noticed that the ADM-2 algorithm with the threshold of 6 suggested by the Agilent company performed quite well, provided that a minimum absolute log_2_ratio for the region is taken into account, such as a MALR > 0.30, as already applied in several studies. In addition, we suggest that also the number of probes concordant should be considered to call the imbalance. For instance, we applied an empirical filter that evaluated if at least one thirds of the probes were above (for gain) or below (for loss) a certain cutoff value, here chosen to be +0.5 and −0.8, finding that the chance to discriminate between true and false calls was greatly improved, especially with the ADM-2 detection algorithm.

A more stringent threshold value for the ADM-2 algorithm did not seem to improve the detection of true imbalances, while, conversely, was at risk of missing the call of several possibly true variants. The same can be said for the Fuzzy Zero algorithm. It is useful for large regions that however are easily detectable as false positive also by the visual inspection of the samples profile.

Very evident large calls, clearly visible at visual inspection and with a neat discrimination above the baseline, were identified with a high level of confidence. They were also easily replied in replicates, including very low quality replicates. This happened for instance for the loss and gain CNVs we included as controls and for two Down syndrome samples. Similarly, the first studies that investigated the reliability of aCGH were mostly based on this sort of gold standard and achieved a complete or almost complete concordance. It remains to explain why other smaller calls seem to be less evident. One possibility is that smaller calls rely on a small number of probes and are therefore more dependent on local probes quality. In addition, problematic regions (such as those closed to centromere) had variability: the *RET* region, known to be difficulty at amplification and screening, was the one to show most false results. In addition, mosaicism cannot be excluded to explain this observation.

It has been suggested that an additional source of variability is given by bench bias [17]. We did not presently investigate such a matter, but from our observation we cannot exclude that correlation and concordance in aberrations calls is dependent on the array and time at which the experiment has been carried out.

Finally, it has also been shown that the fluorescent dyes commonly used in array CGH, fundamentally the red dyes, are sensitive to ozone, and that ozone has a very strong effect on array data especially during the post-hybridization step [37]. We did not specifically address the possibility of false results in this context; however, the arrays were washed and dried under laminar flow hoods in a semi-darkness environment to reduce ozone exposure. Accordingly, we did not observe a difference in gain/loss ratio among the different groups of CNVs (likely, possible, unlikely, true or not true), with the exception of the variants defined as unlikely, basically due to CNVs bigger than 5 kb. The four unlikely true variants (thus possible false negative) were all gains (red dyes predominant) bigger than 5 kb, while among the eight not true variants (possible false positive), the three variants smaller than 5 kb were all gains, and the five variants bigger than 5 kb were all losses. Thus, we cannot exclude that ozone has affected our data, but if this is the case it seems to affect less the smaller aberrations.

In accordance with the impression we got by visual inspection, we have estimated high false positive and false negative rates for the software calls, though these figures need to be taken cautiously. Indeed, we had some concerns given to the fact that few variants detected were then excluded by alternative methods (such as three unlikely and one likely losses and gains excluded by qPCR) and, on the other hand, we were able to detect variants missed by the software and that were successively confirmed to be true (such as three likely losses and gains confirmed by qPCR). Similarly, a deletion found in a sample for which we had two additional replicates, was also detected on the bad quality replicate but not on the good one. In addition, we found two true aberrations in a sample that was in triplicate that were not detected in the best quality replicate and would have thus gone undetected.

Therefore, our observations strengthen the need to validate the results by means of other techniques, among which qPCR is particularly recommended. A priori use of a combined oligo aCGH and SNP array might have avoided some false results, too. Supposed deletions could have been excluded based on heterozygote genotypes in the same region, for instance. However, the non-uniform distribution of informative SNPs throughout the genome might nullify such advantage in specific regions, especially in the case of small CNVs.

Most of the observations reported here come from visual classification, thus lacking of a proved gold standard. Nevertheless, our study provides a confirmation that the high density design of aCGH does not generate more noise than lower density designs and, in addition, does reach a better resolution, with the finding of validated imbalances smaller than 5 kb. In conclusion, our design proved to be reliable, provided that some filters are applied such as MALR ≥ 0.3. We also propose an additional filter, treshhold_e_ > 0.33, which takes into account the proportion of probes with log_2_ratios exceeding suggestive values for gain or loss. In any case, besides the use of additional filters, we would like to stress the importance of paying a great attention to the observation of the samples profiles and the necessity of validating the imbalances detected.

## 4. Materials and Methods

### 4.1. Microarray Design

The sample was constituted by 59 Italian sporadic HSCR patients, six of whom carrying known chromosomal aberrations at the karyotype level: three chromosome 21 trisomies, an invdup(22)(q11) and two interstitial deletions in 10q11.21. The clinical features, selection and processing of the samples are described elsewhere [35].

We have designed a high-density custom array (8X15K SurePrint G3 Human Kit, Agilent Technologies, Santa Clara, CA, USA) through the Agilent eArray web portal. Genomic DNA (test) and sex-matched controls (Promega, Madison, WI, USA) were labelled and hybridized following the protocols provided by the manufacturers. Spot intensities were processed by Agilent Feature Extraction software and the text file outputs were imported into Agilent Genomic Workbench v. 5.0.14 software (Agilent Technologies, Santa Clara, CA, USA) distributed by the vendor.

The microarray consisted of 8333 probes at a high density, selected to cover 20 HSCR candidate genes as described elsewhere, and 3130 probes scattered along the genome, with a probe density of around 1 probe every 900 kb, that constituted the backbone together with 1262 normalization probes (13 of which located in the selected regions), 301 probes replicated five times (1505 probes), and 1482 control probes provided by Agilent (Table 4).

Gene and locus positions are based on the Human Genome GRCh37 (hg19) assembly of UCSC genome browser [38].

### 4.2. Data Analysis and Structural Variant Detection

To investigate genomic imbalances, we applied the ADM-2 algorithm. A threshold of 6 was set, as recommended by Agilent, and a more stringent threshold of 8 has also been tried.

We applied the centralization algorithm and the GC correction algorithm, and we repeated the aberration detection call both applying and not applying the Fuzzy Zero algorithm.

Finally, we have considered as aberrant only those regions with a minimum of 2 probes and with minimum absolute average log_2_ratio for region >0.3. In addition, we evaluated the effect of an additional criteria, the threshold_e_ > 0.33, namely that at least one third of probes in the putative imbalanced interval (at least 2 probes in the case of aberrations based on 3 probes) must present log_2_ratio above 0.5 or below −0.8 for gains and loss respectively (based on log_2_(3/2) = 0.58, log_2_(1/2) = −1).

We also evaluated the samples profiles by visual inspection, reviewed by a second well-trained operator. Loci with nearby gain or loss intervals and an intervening region of more than 2 probes were considered two separate CNVs, as well as those differing for 2 probes presenting inconsistent log_2_ratio (opposite direction, that is log_2_ratio < −0.3 for gain and >0.3 for deletions).

### 4.3. Statistical Analysis

The Agilent Feature Extraction (FE) processes the data, calculates signal log_2_ratios, estimates errors, and provides basic QC metrics. In particular, we have evaluated: (i) the DLRSpread (derivative log_2_ratio spread), which is a measure of the log_2_ratio noise for each sample; (ii) the BGNoise (background noise), which is a measure of the background fluorescence for each channel (Red and Green); (iii) signal intensity; (iv) signal to noise; and (v) the reproducibility for each channel. Measures were considered as excellent, good or to be evaluated, based on Agilent’s guideline (Figure 1).

Newly extracted or purified DNA was run on an additional array for 10 samples with bad profiles, which are those with DRLS ≥ 0.3, and for 16 samples arbitrarily selected. Moreover, four samples were replicated three times (two of which had the third replicate of bad quality), and one sample was replicated four times. When more replicates were available, the variants search was performed in the sample with the lowest DRLS, or, in case of very similar DRLS among replicates, in the one with an overall better quality. In any case, aberrations detected were evaluated also in the replicated samples, when available.

The FPR was determined comparing the average number of variant calls in self-self tests with the average number of variant calls for each sample, using three self-self hybridizations of a reference sample, as described by de Smith et al. [10]. We estimated the FNR using replicated experiments as described by Wong et al. and de Smith et al. [9,10] and adjusted their estimates based on BAC array by changing number of clones with number of probes. While they based their estimate on 6 and 4 replicates, respectively, we used both four and three replicates. Of note, if Wong et al. [9] had performed 4 replicates only, some calls detected more than once would have been lost (virtually present in one of the two additional replicates no more present). For this reason, we calculated by permutation the expected number of calls replicated more than twice by Wong et al. [9] in the case of four (or three) replicates, assuming a random distribution on the replicates experiments. We considered aberrant intervals revealed in different experiment as identical if the overlap among probes was ≥0.90.

We also calculated the correlation between replicates and between random sample pairs selected to have similar DLRS (<0.2, ≥0.2 and <0.3 or ≥0.3). We repeated the analysis for both all log_2_ratios and log_2_ratio exceeding the threshold of absolute 0.3 (considering that those with log_2_ratio not different from 0 are not expected to correlate at all) and among all the replicated samples and among only those with DRLS below the two cutoffs of 0.3 and 0.2. Moreover, we investigated whether the number of aberrations called by the software correlated with the number of probes called, the size, or the probe density, excluding from the analysis the aberrations used as controls and considering the aberrations that overlapped the high density probes regions and the genome as belonging to the high density group. We assumed the aberration size to be the mean between the inner and the outer probes called.

Finally, we tried to investigate whether there was an association between the calls obtained with the software under different criteria and the visual inspection results.

### 4.4. Detected Variants Classification and Validation

The visual inspection of the aCGHsample profiles has allowed us to classify the aberrations detected as known (the controls), likely, possible but not convincing (possible), or unlikely (Table 2).

Aberrations were compared with CNVs observed in the normal population and reported in the Database of Genomic Variants (DGV) [39] and with the CNVs reported in the DECIPHER database of phenotypes, v8.7 released [40]. The comparison between different platforms and techniques is tricky, exact boundaries of the aberrations detected by arrays are not known but only assumed to be between the last “normal” probe (outer) and the first “aberrant” probe (inner) and depend on the average coverage. However, we considered aberrations as consistent with those already reported in the databases if they showed an overlap ≥80%, did not differ for more than two probes with compatible log_2_ratios (that is ≥|0.3|), and were of the same kind (gain or loss). The frequency of the CNV or the number of individuals in the database in which the variant is reported was not a selection criterion but is reported.

We arbitrarily selected the most promising regions (those classified as likely and not reported on the DGV database) and those more interesting for us (i.e., on the *RET* gene) for validation with other molecular biology techniques, and parental check, as described elsewhere [35] and reported in Table 2.

We considered as true calls those detected in the controls (already assessed with other techniques or trisomy 21 in patients also affected by Down syndrome), the aberrations confirmed at the validation, the calls detected also on a second replicate and CNVs reported on DGV.

## 5. Conclusions

Our study provides a confirmation that the high density design of aCGH does not generate more noise than lower density designs and, in addition, does reach a better resolution. However, false positive and false negative results are not trivial. For this reason, we suggest that some filters are applied such as the MALR ≥ 0.3 and the treshhold_e_ > 0.33, this latter taking into account the proportion of probes with log_2_ratios exceeding suggestive values for gain or loss. We have also shown the importance of visual inspection of results and the necessity of validating the imbalances detected.

## Figures and Tables

**Table 1:** Log_2_ratios correlations.

Selected Pairs	Groups Comparisons	*N*	All Log_2_ratios	Log_2_ratios > |0.3|	*p*-Value
			Mean *r*	Mean *r*	
All samples	replicated	37	0.18	0.42	1.8 × 10^−9^
	random	37	0.07	0.14	0.0036
	*p*-value		0.004	4.8 × 10^−5^	
Only pairs with at least one excellent quality sample (DLRS ≤ 0.2)	replicated	24	0.23 *	0.53 ^§^	2.01 × 10^−8^
	random	24	0.09 **	−0.17 ^§§^	0.0057
	*p*-value		0.0018	6.8 × 10^−6^	
Pairs with no excellent quality sample (DLRS > 0.2)	replicated	13	0.09 *	0.21 ^§^	0.003
	random	13	0.05 **	0.09 ^§§^	0.1594
	*p*-value		0.2635	0.1492	

* *p* = 0.0069; ** *p* = 0.2320; ^§^ *p* = 0.0009; ^§§^ *p* = 0.2188.

**Table 2:** Aberrations detected.

Sample ID	DLRS	Chromosomal Region (chr:start–end)	CNV Type	# Probes	Detection Algorithm		Fuzzy Zero	Visual Inspection Classification	Reported on DGV	Reported on Decipher	Validated	Replicate	True Variants ^†^
					ADM-2, Threshold 6	ADM-2, Threshold 8							
HSCR000	0.148	9:110381888–110401999	gain	9	Y	Y	Y	likely	N	N	Y	confirmed	yes
HSCR000	0.148	10:43435867–60812533	loss	849	Y	Y	Y	known	N	N	known	confirmed	known
HSCR000	0.148	10:43572551–43573368	gain	3	N	N	N	unlikely	N	N		not confirmed	no
HSCR037	0.120	10:43589687–62786887	loss	544	Y	Y	Y	known	N	N	known		known
HSCR005	0.226	7:84217007–84225649	loss	4	Y	-	-	likely	Y (freq < 1%)	N	Y		yes
HSCR005	0.226	10:43679892–43680816	loss	5	Y	-	Y	likely	N	N	N		no
HSCR005 *	0.226	21:9833187–11096086	loss	4	N	-	N	possible	N	N			unknown
HSCR006	0.276	10:43679612–43680816	loss	6	N	-	-	likely	N	N	N		no
HSCR006	0.276	10:43685614–43715348	gain	78	N	N	-	unlikely	N	N			unknown
HSCR006	0.276	19:5822193–5832504	gain	13	Y	-	-	unlikely	N	N			unknown
HSCR009	0.176	10:43691613–43713132	gain	50	N	N	N	unlikely	N	N			unknown
HSCR009	0.176	19:5825458–5831976	gain	9	Y	Y	Y	unlikely	N	N			unknown
HSCR010 *	0.211	15:20848460–22432687	gain	5	Y	-	-	likely	Y (freq ≥ 5%)	N		not excluded	yes
HSCR014	0.221	8:32532001–32532545	gain	2	Y	-	Y	unlikely	N	N			unknown
HSCR014 *	0.221	10:29939955–30822470	gain	3	Y	-	Y	possible	N	N			unknown
HSCR014 *	0.221	12:80226392–80589429	gain	2	Y	-	Y	possible	N	N			unknown
HSCR014	0.221	22:22417683–23228483	loss	15	Y	Y	Y	likely	Y (freq ≥ 5%)	N			yes
HSCR016	0.117	5:69288477–70309855	gain	3	Y	-	-	likely	Y (freq ≥ 5%)	N		not excluded	yes
HSCR016	0.117	22:25672585–25892401	gain	5	Y	-	Y	likely	Y (freq ≥ 5%)	Y (3 inds.)		not excluded	yes
HSCR018 ^§^	0.172	9:109336464–109348467	gain	6	-	-	-	likely	N	N	Y		yes
HSCR019 *	0.122	1:146638075–147824207	loss	4	Y	Y	Y	likely	N	Y (1q21.1 recurrent microdel)	Y	confirmed with a different size	yes
HSCR033*	0.229	15:21162691–22173977	loss	3	Y	Y	Y	likely	Y (freq ≥ 5%)	N			yes
HSCR036	0.177	22:22781091–23228483	loss	8	Y	Y	Y	likely	Y (freq ≥ 5%)	N			yes
HSCR039	0.217	3:51458492–51665134	loss	62	N	N	-	unlikely	N	N		not confirmed	no
HSCR039	0.217	6:148651353–150170473	loss	52	N	N	-	unlikely	N	N		not confirmed	no
HSCR039	0.217	9:110130442–110370427	loss	99	N	N	-	unlikely	N	N		not confirmed	no
HSCR043 ^§^	0.175	9:109273643–109275694	loss	2	-	-	-	likely	N	N	Y		yes
HSCR045 ^§^	0.271	7:84594683–84607065	loss	6	-	-	-	unlikely	N	N	N		no
HSCR045 ^§^	0.271	8:32597644–32598929	loss	3	-	-	-	likely	N	N	Y		yes
HSCR045	0.271	10:43679612–43680816	loss	6	Y	-	Y	likely	N	N	N		no
HSCR045	0.271	19:5819037–18310693	gain	25	Y	-	-	unlikely	N	N			unknown
HSCR058	0.243	22:18661724–18920001	gain	7	Y	-	Y	unlikely	Y (freq ≥ 5%)	N		not evaluable	yes
HSCR064 *	0.192	15:20848460–22173977	loss	4	Y	Y	Y	likely	Y (freq ≥ 5%)	N			yes
HSCR126	0.176	19:4205366–18310693	gain	26	N	-	-	unlikely	N	N			unknown
HSCR146 *	0.122	15:58257674–59009890	gain	2	Y	Y	Y	likely	N	N	Y		yes
HSCR146	0.122	19:30888070–30891329	gain	2	Y	-	Y	likely	N	N	N		no
HSCR160 *	0.200	15:20848460–22173977	gain	4	Y	-	Y	likely	Y (freq ≥ 5%)	N			yes
HSCR162 *^,§^	0.184	9:43659247–43659512	loss	2	-	-	-	likely	Y (freq ≥ 5%)	N		confirmed with a different size	yes
HSCR181 *	0.150	15:20848460–22432687	loss	5	N	-	-	possible	Y (freq ≥ 5%)	N		not excluded	yes
HSCR181	0.150	21:14629063–48080926	gain	245	Y	Y	Y	known	N	N	known	confirmed	known
HSCR183	0.138	22:22781091–23228483	loss	8	Y	Y	Y	likely	Y (freq ≥ 5%)	N			yes
HSCR195	0.158	9:112078131–112089193	loss	5	Y	-	-	likely	N	N	inconclusive	confirmed with a different size	yes
HSCR217	0.168	16:82200334–82202467	gain	2	Y	-	Y	likely	N	N	Y		yes
HSCR228 ^§^	0.158	22:25672585–25892401	gain	5	-	-	-	likely	Y (freq ≥ 5%)	Y (3 inds.)		not excluded	yes
HSCR231*	0.164	15:21162691–22432687	gain	4	Y	-	Y	unlikely	Y (freq ≥ 5%)	N			yes
HSCR312	0.215	3:50161771–50618134	gain	143	N	-	-	unlikely	N	N			unknown
HSCR312	0.215	4:41748211–41753993	gain	16	N	-	-	unlikely	N	N			unknown
HSCR312	0.215	10:43550696–43621994	gain	196	N	-	-	unlikely	N	N			unknown
HSCR312	0.215	10:43684681–43718450	gain	86	N	N	N	unlikely	N	N			unknown
HSCR312	0.215	14:36983123–36994136	gain	14	Y	-	-	unlikely	N	N			unknown
HSCR312	0.215	19:5821171–5832504	gain	15	N	N	N	unlikely	N	N			unknown
HSCR323	0.253	13:78465278–78484576	gain	30	N	-	-	unlikely	N	N			unknown
HSCR331	0.172	19:5822193–5832928	gain	14	N	-	-	unlikely	N	N		not excluded	unknown
HSCR335 *	0.183	15:20848460–22173977	gain	4	Y	-	-	possible	Y (freq ≥ 5%)	N		not excluded	yes
HSCR335	0.183	22:18628019–18807881	gain	6	Y	-	Y	unlikely	N	N		not excluded	unknown
HSCR335	0.183	22:20345868–20499789	gain	4	Y	-	Y	unlikely	Y (freq ≥ 5%)	N		not excluded	yes
HSCR335	0.183	22:21494163–21704972	gain	5	Y	-	Y	unlikely	N	N		not excluded	unknown
HSCR349	0.220	3:51452049–51647312	loss	59	N	N	-	unlikely	N	N			unknown
HSCR349 *	0.220	7:63449575–75986814	loss	25	N	-	-	unlikely	N	N			unknown
HSCR349	0.220	10:43573685–43574005	gain	2	Y	-	Y	unlikely	N	N	N		no
HSCR374	0.266	10:43473690–43474033	gain	4	Y	-	Y	unlikely	N	N	N		no
HSCR380	0.123	22:16054691–18807881	gain	23	Y	Y	Y	known	N	N	known		known
HSCR380	0.123	22:20345868–20659606	gain	5	Y	Y	Y	unlikely	N	N			unknown
HSCR380	0.123	22:21494163–21704972	gain	5	Y	Y	Y	unlikely	N	N			unknown
HSCR382	0.235	10:43474436–43483543	loss	29	N	-	-	unlikely	N	N			unknown
HSCR382	0.235	10:43630181–43636329	gain	31	N	-	-	unlikely	N	N			unknown
HSCR382 *	0.235	15:20190548–22173977	gain	5	Y	-	-	possible	Y (freq ≥ 5%)	N			yes
HSCR391	0.173	21:14629063–48080926	gain	245	Y	Y	Y	known	N	N	known	confirmed with a different size	known
HSCR403 ^§§^	0.111	4:41746863–41751291	loss	11	N	-	-	likely	N	N	Y		yes
HSCR403 *^,§§^	0.111	9:43659247–43659512	gain	2	Y	-	Y	likely	Y (freq ≥ 5%)	N			yes
HSCR403	0.111	22:18661724–18807881	gain	5	Y	-	-	possible	N	N		not excluded	unknown
HSCR403	0.111	22:21494163–21704972	gain	5	Y	-	Y	unlikely	N	N		confirmed and not excluded	yes
HSCR403	0.111	22:23056562–23228483	loss	3	Y	-	Y	likely	Y (freq ≥ 5%)	N		confirmed with a different size	yes
HSCR409 *	0.139	15:20848460–22173977	gain	4	Y	Y	Y	likely	Y (freq ≥ 5%)	N			yes
HSCR412	0.204	22:20345868–21778882	loss	26	N	-	-	unlikely	N	N		not confirmed	no
HSCR414 *	0.156	15:20848460–22432687	loss	5	N	-	-	possible	Y (freq ≥ 5%)	N			yes
HSCR415	0.195	9:113025039–113029430	loss	2	Y	Y	Y	likely	Y (freq ≥ 5%)	Y (1 ind.) ^‡^			yes
HSCR421 *	0.166	9:43659247–43659512	loss	2	Y	Y	Y	likely	Y (freq ≥ 5%)	N		confirmed	yes
HSCR421	0.166	22:25672585–25892401	loss	5	Y	Y	Y	likely	Y (freq ≥ 5%)	Y (3 inds.)		not excluded	yes
HSCR426 *	0.111	9:43659247–43659512	loss	2	Y	-	Y	likely	Y (freq ≥ 5%)	N		not confirmed and confirmed	yes
HSCR481 *	0.248	5:7656467–8124532	loss	2	Y	-	Y	possible	N	N		not confirmed	no
HSCR481	0.248	19:31954093–31966036	loss	5	Y	-	-	likely	N	N	Y	not evaluable	yes
HSCR481	0.248	21:14629063–48080926	gain	245	Y	Y	Y	known	N	N	known	confirmed	known

^†^ True (yes) = if either already reported on DGV, validated with different methods or confirmed on at least one replicate; (no) if not validated and/or not confirmed on replicate(s); known = selected controls or known chromosomal rearrangements; unknown = not possible to discriminate between true yes or no; * probes not located in the selected high density regions; ^§^ aberration not detected by the software call, but identified by visual inspection; Y = percentage of probes with absolute high log_2_ratio (≥0.5 for gains and ≤−0.8 for loss) above 33.3%; N = percentage ≤ 33.3%, - = not called by the algorithm; ^‡^ deletion reported as CNV with pathogenicity unknown, reported in an individual with aganglionic megacolon (another name for HSCR), intellectual disability and short stature; ^§§^ aberrations assumed as detected because identified in two additional replicates.

**Table 3:** Detection filters comparison.

Comparison Groups	True Calls	Not Confirmed	Unknown	Total	*p*-Value Likely/Possible vs. Unlikely or Called vs. Not Called by the Software *	*p*-Values Threshold_e_ ≥ 0.33 vs. below *
Likely/possible	39	5	4	48	0.0003 ^†^	
Unlikely	4	8	23	35		
ADM-2_th6 ≥ 0.333	35	6	12	53	1.0000	0.0033 ^††^
ADM-2_th6 < 0.333	3	6	15	24		
NO ADM-2_th6 (visual only) *	5	1	0	6		
ADM-2_th8 ≥ 0.333	18	0	3	21	0.5346	0.0001 ^††^
ADM-2_th8 < 0.333	0	4	5	9		
NO ADM-2_th8	25	9	19	53		
Fuzzy ≥ 0.333	28	6	8	42	0.5230	0.2000
Fuzzy < 0.333	0	1	4	5		
NO Fuzzy	15	6	15	36		
Total	43	13	27	83		

True calls include controls, aberrations reported on DGV, aberrations confirmed in at least a replicate and aberrations confirmed at validation. Not confirmed calls include aberrations not confirmed at validation and not found in the available replicate. Unknown includes calls not validated and not reported on DGV, for which a replicate sample was not available and that have not been evaluated in the statistical test. * *p*-value for true vs. not confirmed calls; ^†^ likely/possible have a significantly higher chance of being true than those unlikely; ^††^ the threshold_e_ ≥ 0.33 filter has a better chance to discriminate between true and false calls, significant for the ADM-2 detection algorithm.

**Table 4:** Regions mapped on the aCGH and probe density.

Kind of Probes	Candidate Region	Locus	# of Features *	# of Unique Probes *	Average Space (nt) *
Selected	*RET*	10q11.2	813	8333	312
	9q31	9q31	1824		2501
	9p24.1	9p24.1	142		3521
	*PHOX2*B	4p13	49		508
	*NRG1*	8p12	473		501
	*SEMA3A*/*SEMA3D*	7q21.11	468		2506
	rs12707682		40		500
	6q25.1	6q25.1	714		3501
	21q22	21q22	202		48,297
	3p21	3p21	1141		3503
	19q12	19q12	1085		3502
	*NRTN*	19p13.3	18		806
	16q23.3	16q23.3	714		3501
	*NKX2-1*	14q13	17		812
	*SOX10*	22q13	27		823
	22q11.2	22q11.2	162		49,383
	*ECE1*	1p36.1	103		806
	*ZEB2*	2q22.3	165		923
	*EDNRB*	13q22	112		804
	*GDNF*	5p13.1-p12	42		810
	*EDN3*	20q13.2-q13.3	44		808
Genome			3149	3130	971,074
Replicates			301 × 5 = 1505	301	
Normalization			1262	1262	
Agilent controls			1482		
Total			15,748	13,026	

* Twenty-two probes selected among the high density panel were also included in the normalization set or in the replicates set and are not reported among the # of unique probes selected, but considered for the average coverage. Nineteen probes selected in the rest of the genome had already been selected for the high density regions (10) or already part of the normalization set (9).
